# Structural Design and DLP 3D Printing Preparation of High Strain Stable Flexible Pressure Sensors

**DOI:** 10.1002/advs.202304409

**Published:** 2023-11-12

**Authors:** Xiangling Xia, Ziyin Xiang, Zhiyi Gao, Siqi Hu, Wuxu Zhang, Ren Long, Yi Du, Yiwei Liu, Yuanzhao Wu, Wenxian Li, Jie Shang, Run‐Wei Li

**Affiliations:** ^1^ School of Materials Science and Engineering Shanghai University Shanghai 200072 P. R. China; ^2^ CAS Key Laboratory of Magnetic Materials and Devices Ningbo Institute of Materials Technology and Engineering Chinese Academy of Sciences Ningbo 315201 P. R. China; ^3^ Zhejiang Province Key Laboratory of Magnetic Materials and Application Technology Ningbo Institute of Materials Technology and Engineering Chinese Academy of Sciences Ningbo 315201 P. R. China; ^4^ State Key Laboratory of Advanced Technology for Materials Synthesis and Processing International School of Materials Science and Engineering Wuhan University of Technology Wuhan 430070 P. R. China; ^5^ School of Physics and BUAA‐UOW Joint Research Centre Beihang University Beijing 100191 P. R. China; ^6^ Materials and Manufacturing Futures Institute School of Materials Science and Engineering The University of New South Wales Sydney NSW 2052 Australia; ^7^ College of Sciences Institute for Sustainable Energy Shanghai University Shanghai 200444 P. R. China

**Keywords:** digital light processing 3D printing, finite element simulation, flexible pressure sensors, strain stability

## Abstract

Flexible pressure sensors are crucial force‐sensitive devices in wearable electronics, robotics, and other fields due to their stretchability, high sensitivity, and easy integration. However, a limitation of existing pressure sensors is their reduced sensing accuracy when subjected to stretching. This study addresses this issue by adopting finite element simulation optimization, using digital light processing (DLP) 3D printing technology to design and fabricate the force‐sensitive structure of flexible pressure sensors. This is the first systematic study of how force‐sensitive structures enhance tensile strain stability of flexible resistive pressure sensors. 18 types of force‐sensitive structures have been investigated by finite element design, simultaneously, the modulus of the force‐sensitive structure is also a critical consideration as it exerts a significant influence on the overall tensile stability of the sensor. Based on simulation results, a well‐designed and highly stretch‐stable flexible resistive pressure sensor has been fabricated which exhibits a resistance change rate of 0.76% and pressure sensitivity change rate of 0.22% when subjected to strains ranging from no tensile strain to 20% tensile strain, demonstrating extremely low stretching response characteristics. This study presents innovative solutions for designing and fabricating flexible resistive pressure sensors that maintain stable sensing performance even under stretch conditions.

## Graphical abstract

Flexible pressure sensors are essential for wearable electronics and robotics, offering stretchability, sensitivity, and integration. Existing sensors face accuracy challenges when stretched. This study employs finite element simulation and DLP 3D printing to enhance strain stability in these sensors. Results show a highly stretch‐stable sensor with minimal resistance and pressure sensitivity changes under strain. This study offers innovative solutions for maintaining stable sensing in flexible resistive pressure sensors.

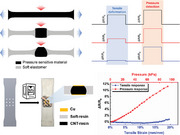

## 1 Introduction

Stretchable flexible pressure sensors, as an emerging technology, have shown vast potential for applications in various fields.^[^ ^1^ ^]^ Their unique flexible design and high adjustability make them an ideal choice for many application scenarios.^[^ ^2^ ^]^ In the healthcare sector, stretchable flexible pressure sensors can be used in devices such as mattresses, seats, and prosthetics to monitor real‐time pressure distribution of patients, prevent pressure ulcers, and provide personalized rehabilitation and prosthetic control solutions.^[^ ^3^ ^]^ In sports training, these sensors can monitor athletes’ force and posture, providing real‐time feedback and guidance to help improve technique and performance.^[^ ^4^ ^]^ In the realm of smart wearable devices, stretchable flexible pressure sensors can be integrated into gloves, wristbands, and head‐mounted devices to enable gesture recognition, virtual reality interaction, motion tracking, and other functions, offering users a more immersive and natural experience.^[^ ^5^ ^]^ Additionally, in automation and robotics technology, stretchable flexible pressure sensors can be utilized for precise object grasping and manipulation, as well as safe collaboration with humans.^[^ ^6^ ^]^ Moreover, in the automotive industry, these sensors can be employed for seat comfort assessment, driver behavior monitoring, and crash safety testing.^[^ ^7^ ^]^ In summary, the application prospects of stretchable flexible pressure sensors are extensive. They will play a significant role in fields such as healthcare, sports, smart wearables, automation, and automotive industries, driving further advancements in related technologies and innovations.

Indeed, flexible pressure sensors face new challenges in their complex operating environments. For instance, these sensors may need to accurately perceive external pressure signals even when subjected to interference strains like bending or stretching. This necessitates that flexible pressure sensors exhibit a high degree of selectivity in their response, meaning they should have low responsiveness to non‐target signals such as stretching and bending while maintaining a higher response to pressure signals. This selectivity is crucial for achieving strain stability in these sensors.

The biggest difference between flexible pressure sensors and rigid sensors is that they can be stretched and bent at will, however, stretching or bending deformation, also makes the flexible pressure sensor and thus affects the perception of pressure. Since most of the existing flexible pressure sensors are not selective for stress/strain, they cannot distinguish what form of external stimulus it is, which seriously affects the recognition of pressure and even causes recognition failure. In order to solve this problem, researchers have also carried out a lot of research work in recent years and proposed two solutions:

One is to improve the anti‐interference ability of flexible pressure sensors by preparing the device into a lattice structure or textile structure that can release tensile/bending strain. For example, Kim et al.^[^ ^8^ ^]^ from the Korea Institute of Basic Science prepared an electronic skin tactile sensor with a fully serpentine grid structure, which was designed to release strain by patterning the structure, and found that the fluctuation of the device parameters at a pressure of 170 kPa was about 60% at a bending radius of ±3 mm. Yun et al.^[^ ^9^ ^]^ from Seogang University, Korea prepared electronic skin tactile sensors by weaving method, utilizing textile structure to release strain, and the fluctuation of the device parameters was about 30% at a pressure of 2N with a stretch of 10 mm. The fabrication of these flexible pressure sensors with serpentine or braided structures involves complex micro and nanofabrication techniques, high fabrication costs, difficulty in realizing high‐volume production, and the problem of low resolution.

Therefore, most researchers are focusing on designing microstructures that can release strains such as stretching/bending on the surface or inside of pressure‐sensitive materials. For example, Park et al.^[^ ^10^ ^]^ from Samsung Advanced Institute of Technology, Korea constructed a continuous pyramid structure on the surface of PEDOT:PSS/86 wt% PUD (poly(3,4‐ethylenedioxythiophene)/poly(styrenesulfonate)/86 wt% polyurethane dispersion) pressure‐sensitive material, and utilized the thinner portion in between pyramids to release tensile strains, and found that the tensile strains were reduced at a 40% tensile strain. tensile strain, the fluctuation of the device parameters at a pressure of 2 kPa was found to be about 75%. Using the same method, Lee et al.^[^ ^11^ ^]^ from Sungkyunkwan University, Korea constructed a continuous concave‐convex structure on the surface of a PEDOT:PSS pressure‐sensitive material, and utilized the concave portion to release the tensile strain, and found that the fluctuation of the device parameters was about 33% at a pressure of 40 kPa under a tensile strain of 30%. Zhao Niobium et al.^[^ ^12^ ^]^ from the Chinese University of Hong Kong prepared graphene@PDMS pressure‐sensitive materials with hollow structures using nickel foam as a template, and released bending strain through the special hollow structure, and found that the fluctuation of the device parameters was about 20% at a pressure of 6 kPa at a bending radius of ±25 mm. To improve the strain stability of flexible pressure sensors, Ryosuke Matsuda et al.^[^ ^1c^ ^]^ designed an Ecoflex‐PDMS‐carbon‐doped porous silicone rubber gradient structure sensor, where the elastic modulus of the materials increased gradually from the outer to the inner layers. This gradient structure allows the strain occurring in the matrix not to affect the carbon‐doped silicone rubber force‐sensitive region at the center, thereby achieving tensile strain stability of the sensor. Yu et al.^[^ ^13^ ^]^ achieved tensile strain stability of the sensor by manipulating the orientation distribution of sea urchin‐shaped nanoparticles through a magnetic field. They prepared a conductive force‐sensitive material with sea urchin‐like spines and arranged the hard conductive particle chains and soft polyurethane (PU) matrix using an external magnetic field, creating anisotropic properties in the force‐sensitive medium of the sensor. The strain‐dependent resistance change of the force‐sensitive medium prepared in this study was only 3.7% at a 600% tensile strain.

However, in these studies, the manufactured force‐sensitive structures are random and lack regularity, making it challenging to explore the correlation between the force‐sensitive structure and strain stability systematically. Consequently, it becomes difficult to extend and apply their methods to the design and fabrication of a broader range of flexible sensors. Exploring pressure‐sensitive materials and device structures with simple processes, large deformation, strong anti‐interference, and high resolution remains an important challenge.

As a high‐precision additive manufacturing technology, digital light processing (DLP) 3D printing can be used to manufacture flexible pressure sensors with complex structures.^[^ ^14^ ^]^ In this work, we adopted finite element simulation optimization and used DLP 3D printing to design and fabricate the force‐sensitive structure of flexible pressure sensors. 18 types of force‐sensitive structures have been designed. This paper systematically studies the influence of different force‐sensing structures on the tensile stability performance of flexible resistive pressure sensors. A design scheme as well as a demonstration device for highly tensile strain‐stable flexible pressure sensors have been successfully obtained. The obtained flexible pressure sensor exhibited a resistance change rate of only 0.76% and a pressure sensitivity change rate of only 0.22% from a no tensile strain state to a 20% tensile strain state, demonstrating extremely low tensile strain response characteristics. This study presents a novel approach for optimizing the design of force‐sensitive structures to achieve flexible resistive pressure sensors that maintain stable sensing performance even under stretching conditions. Furthermore, the study demonstrates the fabrication of these sensors using DLP 3D printing technology.

## 2 Results and Discussions

### 2.1 Principle of Strain‐Insensitive Structure

**Scheme 1:**
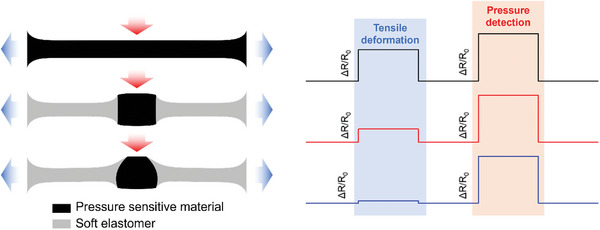
Diagram of structural design to realize tensile strain insensitivity of flexible pressure sensor.

In this study, we achieved high tensile strain stability of the flexible resistive pressure sensor by designing a combined structure that incorporates both soft and hard segments. This approach enhances the sensor's ability to withstand deformation without compromising its performance as shown in **Scheme** **1**. The principle behind this is that the low‐modulus matrix undergoes significant deformation under tensile strain, reducing the influence of tensile strain on the high‐modulus force‐sensitive structure. As a result, it reduces the responsiveness of the sensor to tensile strain.

Scheme 1 depicts the responses of three types of flexible resistive pressure sensors under the condition of the same tensile strain without external force and the same external force but without stretching respectively: A sensor made of pure force‐sensitive material, a flexible matrix embedded with a traditional columnar force‐sensitive structure, and a flexible matrix embedded with an optimized force‐sensitive structure.

For a sensor made of purely force‐sensitive material, the longitudinal axis of the material shrinks when stretched uniaxially (Poisson effect), which produces a similar resistance change signal to the material when the longitudinal axis is compressed. The flexible pressure sensor with a traditional columnar force‐sensitive structure embedded in the flexible matrix exhibits an advantageous characteristic during uniaxial stretching compared to the previous one. The softer substrate will bear most of the stress, resulting in larger deformation, so that the sensor can maintain the integrity of the force‐sensitive structure with a larger modulus, and at the same time have better tensile stability. Consequently, the combination of soft and hard segments can enhance its resistance to interference caused by tensile strain. In the flexible resistive pressure sensor with a flexible matrix embedded with an optimized force‐sensitive structure, the interference resistance to tensile strain is further improved, and the structural design enhances the sensitivity to external force, that is, pressure sensitivity, compared to the cylindrical structure. The specific optimization details will be described in Sections 2.2 and 2.3.

Overall, the primary design concept in this paper is the incorporation of an optimized force‐sensitive structure within a flexible substrate, aiming to create a flexible resistive pressure sensor that remains unaffected by tensile strain. By embedding the optimized force‐sensitive structure into the flexible substrate, the sensor retains its exceptional pressure response even when subjected to stretching or tensile forces.

### 2.2 Parameterized Design of Force‐Sensitive Structure

**Figure 1:**
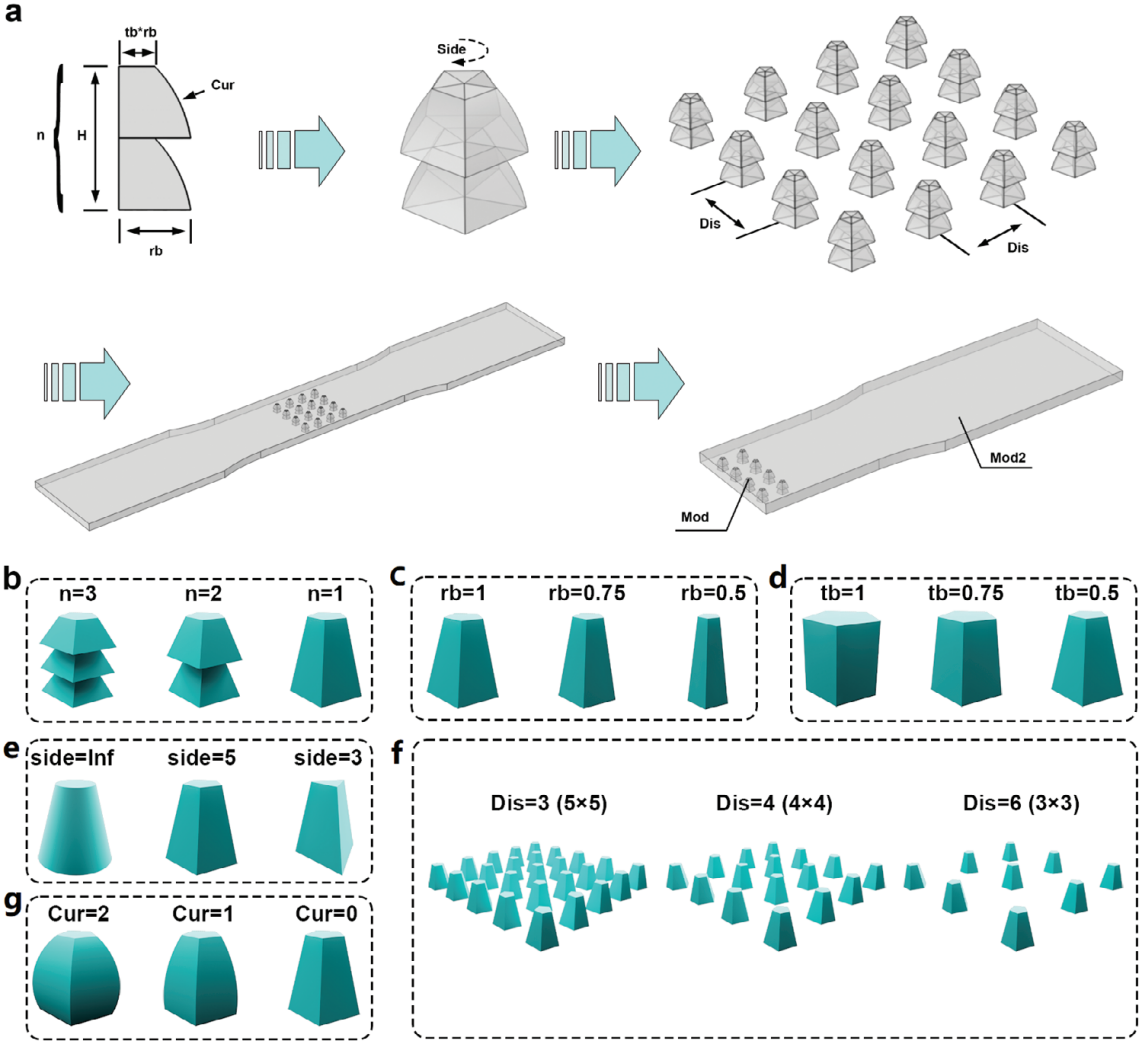
Construction of 3D models of force‐sensitive structures. a) The workflow of parameterized 3D modeling. The corresponding relationship between each parameter and the geometric structure and spatial distribution of the force‐sensitive structure of the sensor: b) parameter n, c) parameter rb, d) parameter tb, e) parameter side, and f) parameter Dis, g) parameter Cur.

**Figure** **1** illustrates the design process of the strain‐insensitive force‐sensitive structure in this study. This process enables parametric design of force‐sensitive structures with comparable performance. Figure 1a shows the workflow of parameterized 3D modeling. The process begins with constructing the cross‐section of the geometric shape, rotating it to obtain three‐ dimensional structures with different numbers of edges, and then arraying the structural units to form a force‐sensitive structure array. Next, the force‐sensitive structure array is combined with a dumbbell‐shaped soft substrate to form the three‐dimensional model of the sensor. The dumbbell‐shaped soft substrate has an array of reserved holes that match the force‐sensitive structure. To simplify the computational load of finite element simulation, the model is divided into half based on symmetry. In the 3D modeling process, it is observed that for a fixed thickness (H) of the whole structure, by varying the number of stacks (n), the bottom edge length of the cross‐section (rb), the ratio of top edge length to bottom edge length (tb), the curvature of the side edge (Cur), the number of sides (Side), and the array spacing (Dis), these 6 geometric parameters, a wide range of common geometric shapes can be obtained, such as cylinders, prisms, cones, hemispheres, etc. Thus, parameterization allows for comparative analysis of the effects of each parameter on the geometric morphology of the force‐sensitive structure, as shown in Figure 1b‐g.

Furthermore, it is known from the derivation of the classical linear elastic equation that the material parameters (elastic modulus/hardness) of the force‐sensitive structure (Mod) and the soft matrix (Mod2) also affect the strain resistance response of the sensor (Figure S1, Supporting Information). Therefore, the material parameters are also considered as variables in the simulation. To achieve the desired effect of strain release in the soft substrate, it is necessary to reduce Mod2 or adjust Mod. However, in actual fabrication, the soft elastic substrate is made of pure photosensitive resin material, and there are no means used to reduce the modulus and hardness of the photosensitive resin other than by adding a hardener, which can increase the modulus and hardness of the force‐sensitive structure, effectively changing the Mod parameter. Therefore, in the simulation, the Mod2 parameter is a fixed value, and simulations are conducted with variations in Mod.

### 2.3 Orthogonal Experimental Design

In this study, an orthogonal experimental design method was used to perform finite element simulations on the 6 geometric parameters and 1 set of material parameters Mod (elastic modulus/hardness) mentioned above. Three levels were chosen for each parameter, represented by 1, 2, and 3, the higher the value, the larger the current parameter. An L_18_(3^7^) orthogonal table was used to arrange the simulation experiments. The orthogonal table and the corresponding specific parameters are detailed in Tables S1 and S2, Supporting Information. In the finite element simulation, the sensor model was subjected to uniaxial stretching of 20%, and the corresponding resistance change was recorded. This study conducted stress‐strain tests using ISO standard tensile specimens to validate the simulation models. Figure S2, Supporting Information presents the simulated values (including hyperelastic and linear elastic models), experimental values, and theoretical values based on the Yeoh hyperelastic model. The comparative results indicate that using the hyperelastic material model for finite element calculations in this study is highly reliable.

**Figure 2:**
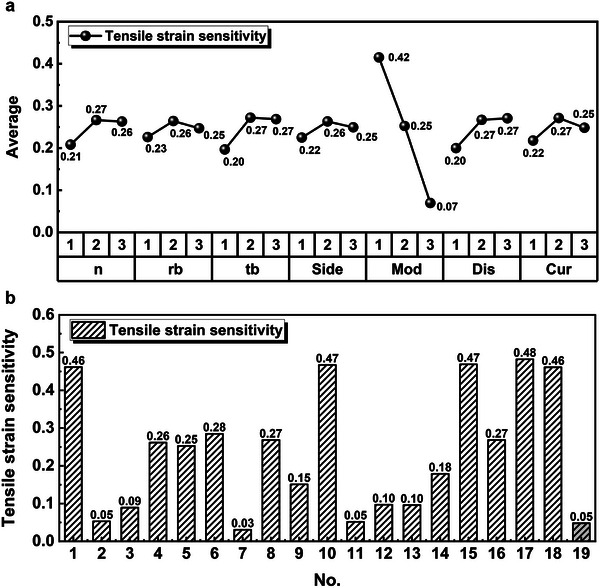
Orthogonal Experimental Results. a) Range analysis for the orthogonal experimental design. b) *T*‐values for the experimental and optimized groups in the orthogonal experimental design.

In order to quantify the concept of tensile strain stability, we next use tensile strain sensitivity to describe the stability of the sensor's external force sensing under tensile strain. The smaller the strain sensitivity, the higher the stability. The tensile strain sensitivity was then calculated as the objective parameter. The range analysis results of the orthogonal experiment and the corresponding strain sensitivity distributions for each group are shown in **Figure** **2**. The range of strain sensitivity in the figure can reflect the degree of influence of parameters on performance.

From Figure 2a, it can be observed that the Mod parameter has the largest influence on the strain sensitivity of the sensor. The relationship between the Mod parameter and the strain sensitivity follows the pattern that a larger Mod value leads to a smaller strain sensitivity, which is consistent with the inference from the linear elastic equation. In the range analysis of the orthogonal experiment, the influence of other parameters on the strain sensitivity of the sensor is relatively small. This might be due to the limited number of levels (three levels) for some parameters and the wide range of values for these variables during the selection of parameter levels. As a result, it becomes challenging to highlight the impact of variables on sensor performance with such a wide variability. Therefore, it is necessary to conduct the single‐variable simulations as presented in the following sections.

By combining the lowest level values of each parameter from the range analysis, the optimized structure group, referred to as the 19^th^ model structure, was obtained. Figure 2b shows a column chart of the strain sensitivity corresponding to the 19^th^ model structure and the remaining 18 groups of structures. It can be observed that although the strain sensitivity of the 19^th^ structure is slightly higher than that of the 7^th^ structure, it is comparable to the 2^nd^ and 11^th^ structures. This indicates that the orthogonal experimental design has achieved certain optimization effects.

### 2.4 Single‐Variable Experiment

**Figure 3:**
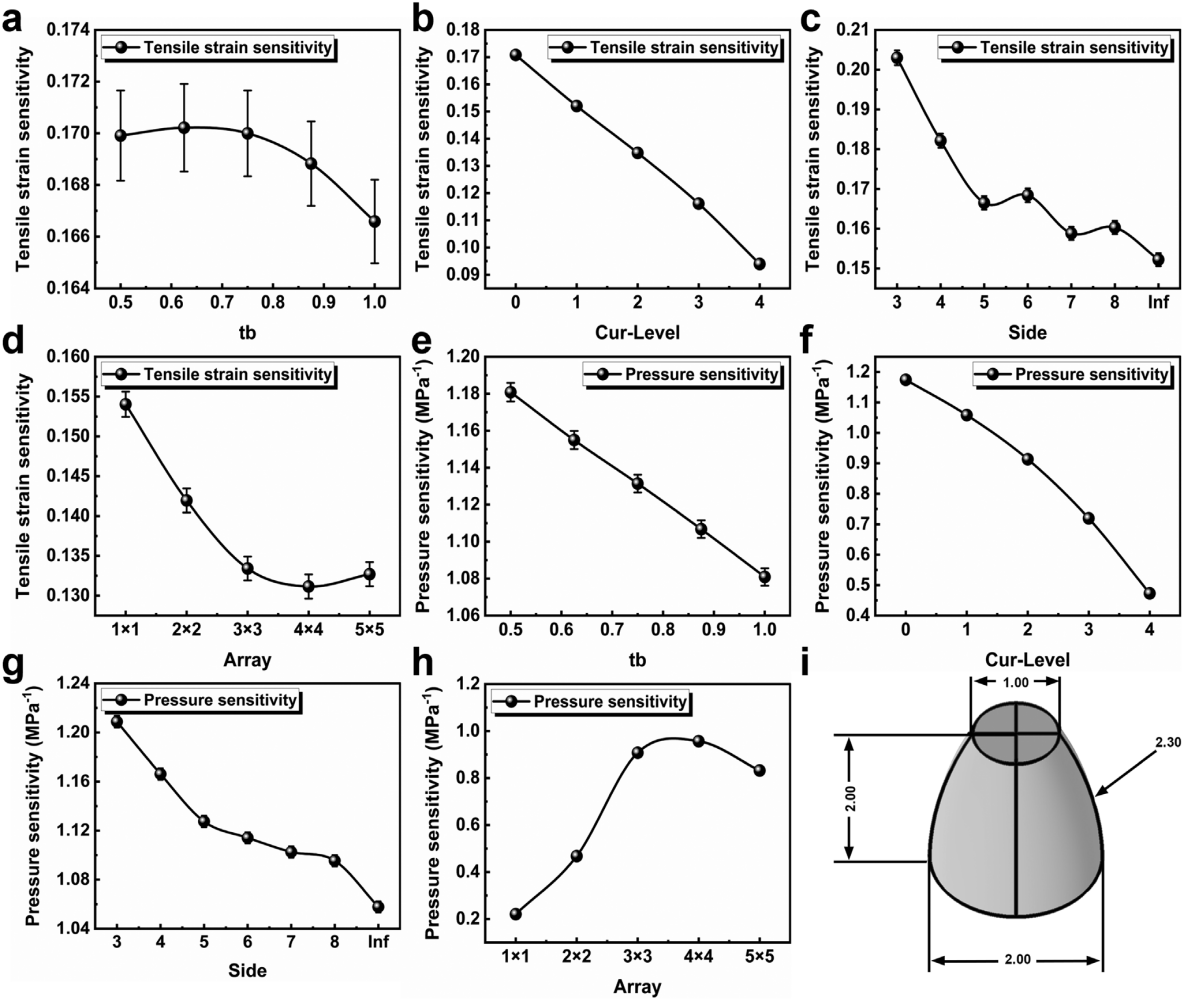
Impact of parameters on strain sensitivity and pressure sensitivity of the sensor. a) The top‐to‐bottom ratio, b) curvature, c) side edge count, and d) array density were studied for their influence on the strain sensitivity of the sensor. e) The top‐to‐bottom ratio, f) curvature, g) side edge count, and h) array density were studied for their influence on the pressure sensitivity of the sensor. i) The finite element optimized force‐sensitive structural model (units in mm).

The optimized structure obtained through orthogonal experimental design did not achieve the lowest strain sensitivity, but it has been determined that the Mod parameter should have a larger value. Additionally, due to the limitations of 3D printing precision, the parameters *n* and *rb* were fixed at 1 and 1 mm, respectively, and further systematic exploration was conducted on the parameters tb, Cur, Side, and Dis (Array), as shown in **Figure** **3**. The influence of each parameter on the tensile strain sensitivity is explored one by one by the single variable method. The values of the independent variable and fixed parameter values in the univariate experiment are shown in **Table** **1**.

**Table 1:** Preparation of flexible pressure sensors with different force‐sensitive structures and materials.

Independent variable	Variable value	Fixed parameter value
Tb	0.5	Side = Inf, Dis = 6 mm (Array = 3 × 3), Cur = 0
	0.625	
	0.75	
	0.875	
	1	
Cur (*C*_max_ = 941.176 m^−1^)	0	*tb* = 0.5, Side = Inf, Dis = 6 mm (Array = 3 × 3)
	3/13 C_max_	
	6/13 C_max_	
	9/13 C_max_	
	12/13 C_max_	
Side	3	*tb* = 0.5, Dis = 6 mm (Array = 3 × 3), Cur = 217.195 m^−1^
	4	
	5	
	6	
	7	
	8	
	Inf	
Dis (Array)	20 mm (1 × 1)	*tb* = 0.5, Cur = 217.195 m^−1^, Side = Inf
	12 mm (2 × 2)	
	6 mm (3 × 3)	
	4 mm (4 × 4)	
	3 mm (5 × 5)	

Figure 3a–d depicts the trends of tensile strain sensitivity with respect to the parameter values. As tb, Cur, and Side increase, the strain sensitivity of the sensor decreases. This change may be related to the tendency of the force‐sensitive structure towards a spherical shape. On the other hand, as the array density increases, the strain sensitivity initially decreases and then increases, indicating that appropriately increasing the array density can reduce the strain resistance response of the sensor. However, when the force‐sensitive structure becomes too densely distributed, the ability of the low‐modulus matrix to release strain is weakened, leading to an increase in tensile strain sensitivity.

Figure 3e–h illustrates the trends of pressure sensitivity with respect to the parameter values. As tb, Cur, and Side increase, the pressure sensitivity of the sensor decreases, which is unfavorable for accurate pressure sensing. As for the array density, the pressure sensitivity of the sensor first increases and then decreases.

Figure 3i presents the geometric parameters of the optimized force‐sensitive structure, which exhibits an overall shape of a laterally expanded truncated cone. The selection of optimized parameters considers the relative changes in both strain sensitivity and pressure sensitivity, To achieve our objective of maximizing pressure sensitivity while minimizing tensile strain sensitivity, we adopt a comprehensive approach. By calculating the ratio of pressure sensitivity to tensile strain sensitivity, denoted as P/S, we can effectively evaluate the sensor's performance. A higher P/S value indicates a more desirable outcome, as it signifies superior pressure sensitivity relative to its sensitivity to tensile strain (please refer to Figure S3, Supporting Information). For instance, the increase in the tb parameter has a relatively low impact on strain sensitivity (≈2% variation), but it has a significant effect on pressure sensitivity (≈8%). The optimized structure has a bottom circular diameter of 2 mm, a height of 2 mm, a top circular diameter of 2 mm, and a side curvature of 434 m^−1^ (corresponding to an inner diameter of 2.30 mm).

We also conducted simulation calculations with different layout patterns, including circular arrangement, star arrangement, staggered arrangement, and a layout where the matrix arrangement is rotated by 45 degrees (Figure S4, Supporting Information). In these structural layouts, we ensured that the shortest distance between sensitive units was the same. We separately calculated the strain sensitivity and pressure sensitivity of sensors with these patterns. Based on the comparison of simulation data, the differences in strain sensitivity performance among these layout patterns were not significant. The circular arrangement, star arrangement, and staggered arrangement showed slightly higher strain sensitivity compared to the matrix arrangement (Δ*S*_tensile_ < 0.7%). However, there was a slightly larger variation in pressure sensitivity, with the circular arrangement, star arrangement, and staggered arrangement slightly lower than the matrix arrangement (Δ*S*_pressure_ ≈1.6%).

In contrast, the changes in parameters such as tb, Cur, and Side caused much larger variations in strain sensitivity (Δ*S*_tensile_) and pressure sensitivity (Δ*S*_pressure_) than the variations in sensor performance caused by layout patterns. Therefore, in this study, we used the conventional matrix‐type layout pattern. Additionally, this result suggests that the layout pattern of the prepared sensor array can be adjusted according to the specific application scenario without significantly affecting its performance.

### 2.5 Performance Characterization

**Figure 4:**
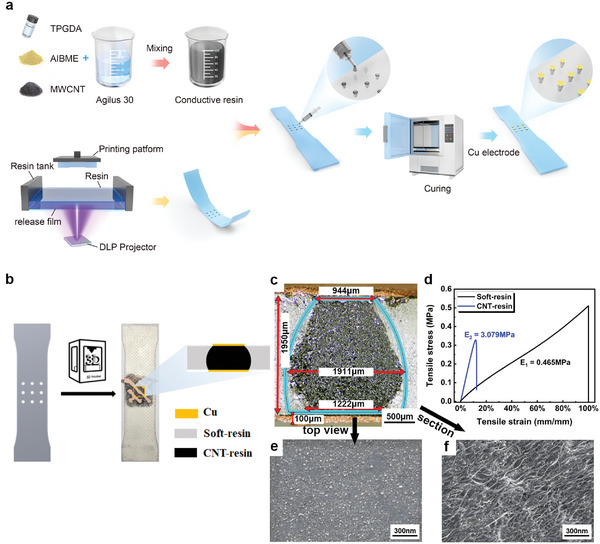
Manufacturing process of DLP 3D printed flexible pressure sensor with high strain stability. a) Process flowchart for fabrication. b) Schematic diagram of the sensor structure. c) Optical magnification image of the sensor's force‐sensitive structure. d) Stress‐strain curves of the sensor's force‐sensitive structure and matrix material. e) SEM image of the top surface of the sensor's force‐sensitive structure. f) Cross‐sectional SEM image of the sensor's force‐sensitive structure.

Based on the influence patterns of force‐sensitive structures on the tensile strain sensitivity and pressure sensitivity of the sensor summarized from finite element simulations, flexible pressure sensors with high strain stability were further fabricated using DLP 3D printing technology. **Figure** **4a** illustrates the process of fabricating flexible pressure sensors through 3D printing technology. Firstly, an elastic matrix with microholes of the same size as the force‐sensitive structure was fabricated using a 3D printer. Then, the microholes were filled with conductive resin slurry, and after thermal curing, the sensor fabrication was completed by connecting the testing circuit. The selection of photosensitive resin and the composition ratio control of the conductive slurry are detailed in the Supporting Information.

Figure 4b,c show the macroscopic structure of the fabricated sensors. We conducted measurements on the cross‐section of the force‐sensitive structure (Figure 4c). The blue contour lines correspond to the cross‐section of the designed structure. In comparison to the upper part of the structure, the printed structure near the bottom exhibits significant differences from the design structure. One possible reason for this is that during the printing process, UV light passing through the upper transparent resin causes structural changes in the bottom due to secondary exposure. We compared the finite element simulation results of the actual fabricated structure with the designed structure model (Figure S5, Supporting Information). In comparison to the designed structure, the actual fabricated structure shows a slight reduction in strain sensitivity (from 0.134 to 0.122), suggesting that the actual structure may have higher strain stability than the designed one. In terms of pressure sensitivity, the actual structure exhibits a slight decrease compared to the designed structure, primarily due to an increase in initial resistance. The absolute value of resistance change differs only by −4% (from 2.979 to 2.884 Ω), while the initial value changes by 13% (from 38.814 to 44.279 Ω). The cumulative effect of these changes results in a 14% reduction in pressure sensitivity for the actual structure compared to the designed structure. Therefore, the structure fabricated in this study can be considered a variant based on the designed structure, and its sensor performance is approximately similar to the designed structure.

Figure 4d–f shows the microscopic morphology of the fabricated sensors. MWCNTs are uniformly distributed in the resin, forming a conductive network that constitutes the force‐sensitive structure. The modulus of the sensor's force‐sensitive structure (3.079 MPa) is significantly higher than that of the matrix (0.465 MPa), satisfying the design of a soft‐hard segment structure.

**Figure 5:**
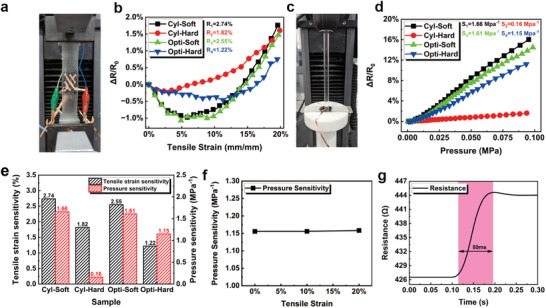
Characterization of force‐electrical performance of sensor samples. a) Uniaxial tensile schematic diagram. b) Tensile strain‐resistance response curve of the samples. c) Uniaxial compression schematic diagram. d) Pressure‐resistance response curve of the samples. e) Tensile strain resistance variation rate and pressure sensitivity of the samples. f) Pressure sensitivity of Opti‐Hard samples at different strains. g) Response time of Opti‐Hard Samples to pressure.

To validate the improved tensile strain stability of the force‐sensitive structure in the flexible pressure sensor achieved through finite element optimization, and to investigate the effect of modulus variation on stability, four sets of samples were prepared, as shown in **Table** **2**. The electromechanical properties of these four samples were characterized, including tensile strain‐resistance response, pressure‐resistance response, tensile strain resistance variation rate and pressure sensitivity, response time, and so on, as shown in **Figure** **5**.

**Table 2:** Preparation of flexible pressure sensors with different force‐sensitive structures and materials.

No.	Force‐sensitive structure	Additives
Cyl‐Hard	Cylindrical	TPGDA
Cyl‐Soft	Cylindrical	Equal mass of A30‐A
Opti‐Hard	Optimized	TPGDA
Opti‐Soft	Optimized	Equal mass of A30‐A

Figure 5a shows the resistance change rate caused by tensile strain under stretching. Figure 5c shows the resistance change rate caused by sensing pressure by the sensor without tensile strain. Figure 5a,b illustrate the resistance changes of the four fabricated sensors during the 0–20% tensile strain process. The corresponding resistance change rates were 2.74% (Cyl‐Soft), 1.82% (Cyl‐Hard), 2.55% (Opti‐Soft), and 1.22% (Opti‐Hard). Comparing the Cyl‐Hard and Cyl‐Soft samples, it can be observed that the softer force‐sensitive material exhibited a larger resistance change under tensile strain, consistent with the two Opti samples. Furthermore, comparing the Cyl‐Hard and Opti‐Hard samples, it can be seen that the Opti‐Hard sample, which has an optimized design structure, demonstrated a lower resistance response to tensile strain. However, it is difficult to distinguish between the two sets of Soft samples in terms of their response to tensile strain. This observation may suggest that a higher hardness of the force‐sensitive structure is a prerequisite for improving strain stability.

We noticed that in Figure 5b, the relative resistance change of the sensor exhibits a decreasing trend in the early stages of tensile strain. One possible explanation for the phenomenon of resistance decreasing and then increasing during the stretching process of the sensor is that carbon nanotubes (CNTs) in the sensor form a resistance network similar to Figure S11, Supporting Information. Simplistically, this equivalent circuit consists of a main path and a branch path. In the initial stages of stretching, both the main path and the branch path remain connected. Due to the Poisson effect, the distance in the vertical direction of the conductive network shortens, resulting in a decrease in resistance. However, when the stretching exceeds a certain degree, the branch path disconnects from the main path, causing an overall increase in resistance in the circuit. Therefore, during the actual stretching process, the phenomenon of resistance decreasing and then increasing occurs. This effect is challenging to model through finite element simulations, so the finite element simulation in this study did not introduce this effect and only considered the influence of strain on resistance.

Figure 5c,d depict the response curves of the four fabricated sensors to compressive stress ranging from 0 to 100 kPa, with corresponding pressure sensitivities of 1.66 MPa−1(Cyl‐Soft), 0.16 MPa−1 (Cyl‐Hard), 1.61 MPa−1 (Opti‐Soft), and 1.15 MPa−1 (Opti‐Hard). A comparison between the Cyl‐Hard and Cyl‐Soft samples reveals that increasing the hardness of the force‐sensitive structure significantly reduces the pressure sensitivity of the device. Although the pressure sensitivity changes of the two Opti samples are relatively small, they exhibit consistent trends. Comparing the Cyl‐Hard and Opti‐Hard samples demonstrates that the pressure sensitivity of the device can be greatly improved through the optimized design of the force‐sensitive structure. The pressure sensitivities of the Cyl‐Soft and Opti‐Soft samples are relatively close, while the Opti‐Hard sample shows a significant deviation from the Cyl‐Soft sample, indicating that although the structural design can enhance the sensitivity of the sensors prepared in this study to some extent, it is still challenging to counteract the sensitivity reduction caused by the hardening of the force‐sensitive material. Figure 5e summarizes the performance parameters of pressure sensitivity and strain sensitivity for the four samples, with the Opti‐Hard sample, featuring a structurally optimized and higher hardness, exhibiting the lowest strain sensitivity and higher pressure sensitivity. Figure 5f further demonstrates the variation of pressure sensitivity of the Opti‐Hard sample under 0–20% tensile strain, indicating minimal changes in pressure sensitivity under different tensile strain conditions. Figure 5g presents the response speed of the sensor to pressure signals, showing that the sensor has a response time of ≈80 ms for instantaneous external loading, demonstrating its real‐time response capability.

**Figure 6:**
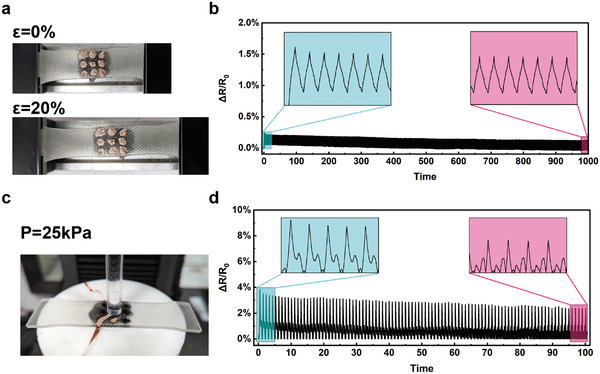
Cyclic stability test of flexible pressure sensor. a) Schematic diagram of tensile cyclic testing. b) Resistance change rate curve during tensile cyclic testing. c) Schematic diagram of compression cyclic testing. d) Resistance change rate curve during compression cyclic testing.

In addition, to evaluate the stability and durability of the sensor, 1000 cycles of 20% tensile strain and 100 cycles of 25 kPa pressure were performed on the samples. **Figure** **6**a,b shows that after 1000 cycles of tensile strain, the resistance response remained consistent with the initial state, indicating that the sensor has a longer operational lifespan. Figure 6c,d reveals that after 100 cycles of compression testing. Figure 6d exhibits inconsistency in the waveforms at the beginning and end of the cycling. During the initial cycles, the force‐sensitive structure maintains relatively good resilience. However, after multiple compression cycles, there is a certain degree of reduction in resilience. At the end of one compression cycle, the deformation of the force‐sensitive structure has not fully recovered, and it undergoes further deformation under the compressive stress of the next compression cycle. Therefore, in the later cycles, there is a deviation in the resistance waveform of the sensor compared to the beginning, primarily due to this cumulative effect where the deformation is not fully restored between cycles.

**Table** **3** lists the performance of similar flexible pressure sensors, and the flexible pressure sensor fabricated in this study exhibits the lowest strain resistance response and pressure sensitivity variation.

**Table 3:** Performance of flexible pressure sensors reported in related studies.

Ref.	Resistance/Resistivity (*ε* = 0%)	Resistance/Resistivity (*ε* = 20%)	Δ*R*/*R*_0_ ^a)^ (0–20%)	Δ*S*_p_/*S*_p0_ ^b)^ (0–20%)	Measurement range
			%	%	kPa
[10]	1053 Ω/□	1111.5 Ω/□	5.55	49.18	0–8
[15]	10.2 kΩ	10.68 kΩ	4.70	3.60	0–4
[16]	–	–	2.20	2.98	0–6
[17]	210 kΩ	246 kΩ	17.14	1.06	200–1000
[18]	–	–	24.60	6.21	0–1000
[19]	14.5 Ω	26.0 Ω	79.31	18.48	0–25
[20]	–	–	334	7.89	0–60
[21]	–	–	5.80	2.49	0–10
[22]	6.44 MΩ	6.32 MΩ	1.86	14.23	0–32
This work	515 Ω	518.9 Ω	0.76	0.22	0–100

^a)^ Δ*R*/*R*_0_ = (*R*‐*R*_0_)/*R*_0_;

^b)^ Δ*S*_p_/*S*_p0_ = (*S*_p_‐*S*_p0_)*S*_p0_, *S*_p_ is pressure sensitivity.

### 2.6 Applications

**Figure 7:**
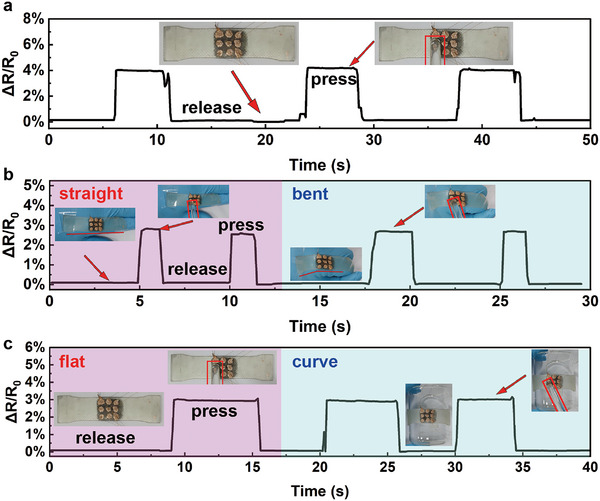
Mechano‐electric properties of flexible pressure sensors in different service environments. a) Response graph of the sensor to pressing on a plane. b) Response graph of the hand‐held sensor to pressing with the finger joint extended and bent. c) Response graph of the sensor to pressing on a plane and on the curved surface of a beaker.

**Figure** **7** illustrates the application of the high‐strain‐stable flexible pressure sensor under different strain conditions, demonstrating that the flexible pressure sensor prepared in this study exhibits low responsiveness to varying strain conditions (consistent baseline) and provides similar responses to external pressure under different strain conditions. Figure 7a–c shows the resistance response curves of the sensor when pressed on a flat surface, when the sensor is subjected to finger joint extension‐flexion, and when the sensor is placed on a flat‐curved surface, respectively. The resistance response of the sensor to external pressing stimuli under different strain conditions can be observed: The sensor exhibits high stability and minimal resistance variation under tensile and bending strain conditions. The sensor demonstrates excellent pressure response characteristics on different surfaces and under various strain conditions. These results indicate that the sensor structure optimized through finite element simulation possesses good strain stability and selective response characteristics to both tension and pressure stimuli.

**Figure 8:**
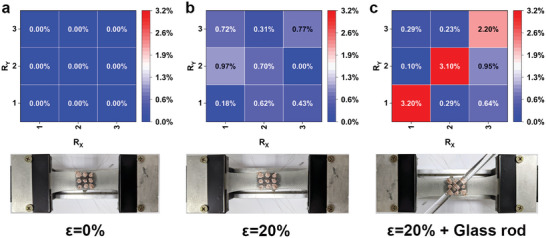
Resistance relative change and corresponding schematic of the sensor array under conditions of a) ε = 0%, b) ε = 20%, and c) ε = 20% with a glass rod placed.

**Figure** **8** showcases the application of the high‐strain‐stable flexible pressure sensor in a 3 × 3 array configuration, indicating that the flexible pressure sensor designed in this study possesses the capability for array integration and demonstrates high strain stability within the sensor array. When the sensor is subjected to 0% to 20% strain (Figure 8a,b), the resistance variation of the sensor units is low, indicating a low strain response. When a glass rod is placed on the sensor under 20% strain (Figure 8c), the sensor is capable of detecting the pressure exerted by the glass rod. This confirms that the high‐strain stable flexible pressure sensor array exhibits selective response capabilities to both tensile strain and pressure stimuli.

## 3 Conclusion

This work systematic study of how force‐sensitive structures enhance the tensile strain stability of flexible resistive pressure sensors. 18 types of force‐sensitive structures have been investigated by finite element design, simultaneously, the modulus of the force‐sensitive structure is also a critical consideration as it exerts a significant influence on the overall tensile stability of the sensor. Through finite element optimization, a satisfactory optimal force‐sensing structure that enables the flexible pressure sensor to maintain excellent force‐sensing performance under tensile strain is obtained: A laterally expanding truncated cone shape. The sensor is fabricated using DLP 3D printing technology to print an elastic substrate, which is then infused with a formulated conductive resin slurry to create a high‐strain stable flexible pressure sensor. Experimental and simulation results are used to summarize the effects of the geometric morphology and material modulus of the force‐sensitive structure on the strain sensitivity and pressure sensitivity of the sensor. When this structure is stretched, the low‐modulus flexible substrate undergoes significant deformation while the high‐modulus force‐sensitive structure is less affected, thereby maintaining a stable resistance. As a high‐strain stable flexible pressure sensor, it exhibits a resistance variation of only 0.76% and a pressure sensitivity change of only 0.22% under 20% strain. Due to its high‐strain stability, this sensor holds great potential for applications in the field of wearable electronics. The application of the sensor array, which exhibits a highly selective response to strain and pressure, validates the suitability of this structure as a high‐strain stable flexible pressure sensor in practical applications. This work, combining structural design and DLP 3D printing, provides inspiration for the development of bulk‐structured flexible pressure sensors.

## 4 Experimental Section

### Materials

Agilus 30 resin was purchased from Stratasys (Eden Prairie, MN, USA). X23 and X29 resins were purchased from Zhongshan Huayu Yuanxing Electronic Technology Co., Ltd. (Guangdong, China). F39‐T resin was purchased from Dongguan Shenshuo Technology Co., Ltd. (Guangdong, China). Are3D resin was purchased from Shenzhen Pandora 3D Technology Co., Ltd. (Guangdong, China). Multi‐walled carbon nanotubes ((MWCNTs, 99%) were purchased from Shenzhen Suiheng Technology Co., Ltd. (Guangdong, China). Tri(propylene glycol) diacrylate (TPGDA, 90%), dimethyl 2,2’‐azobis(2‐methylpropionate) (98%), and isopropanol (95%) were purchased from Shanghai Aladdin Bio‐Chem Technology Co., Ltd. (Shanghai, China). Conductive copper paste was purchased from Suqian Teding Shangwu Co., Ltd., Suqian, China. All chemicals utilized were of analytical grade and did not require further purification.

### Finite Element Simulation

Finite element simulations were performed using COMSOL Multiphysics 6.1. The model utilized the Yeoh hyperelastic material model, and the parameters of the Yeoh model were fitted to the uniaxial tensile test data (ISO 37:2017). The model was subjected to uniaxial tensile and compression tests, and the changes in resistance with model deformation were recorded.

### Preparation of Conductive Resin

A certain amount of Agilus 30 resin was mixed with Dimethyl 2,2’‐Azobis(2‐ methylpropionate) (AIBME) powder. The mixture was placed in a planetary mixer (ZYMC‐200 V, Suzhou Zhongyi Precision Technology Co., Ltd., Jiangsu, China) and stirred to obtain the heat‐curable A30‐A slurry. MWCNTs were used as the conductive filler, and a certain amount of TPGDA was added as a hardener. The MWCNTs and TPGDA were mixed with the A30‐A slurry to obtain the A30‐A‐MC‐T slurry.

The planetary mixer was operated in multiple stages. Firstly, it was run at 600 rpm for 30 s for initial mixing. Then, it was run at 1500 rpm for 60 s while maintaining a vacuum of −100 kPa (relative to atmospheric pressure) inside the chamber. Finally, the vacuum was maintained at −100 kPa, and the mixer was run at 1000 rpm for 60 s.

### DLP 3D Printing of the Matrix

The designed 3D model file was sliced into 50 µm layers and imported into a DLP 3D printer (AUTOCERAL, Beijing Shiwei Technology Co., Ltd., Beijing, China). The flexible resin Agilus 30 was cured with UV exposure at an intensity of 20 mW cm^−2^ for 1.2 s per layer with a layer thickness of 50 µm. The printed product was removed from the build platform and immersed in isopropanol solution for 5 min (25 kHz) in an ultrasonic bath. After drying, the printed matrix was ready for use.

### Fabrication of Sensor Devices

The prepared conductive slurry was injected into the cavity of the 3D‐printed matrix using a syringe. A glass slide was then used to seal the cavity, and the assembly was heated at 80 °C for 1 h to obtain the desired structure of the flexible pressure sensor. Conductive copper paste was applied to the top and bottom surfaces of the force‐sensitive structure to create electrodes. After curing the copper paste, the sensor device was ready for connection to the testing circuit.

### Electrical Performance

The resistance was measured using the four‐wire method with a DC current source (Keithley 6221, Cleveland, OH, USA) and a nanovoltmeter (Agilent 34420A, Santa Clara, CA, USA). Copper sheets were connected to both ends of the sample, linking it to the current source and voltmeter. The resistance change of the linear‐bunch‐structured stretchable conductor was observed using a data acquisition system while subjecting it to stretching using the stretching machine (Instron 5943).

## Conflict of Interest

The authors declare no conflict of interest.

## Supporting information

Supporting information

## Data Availability

The data that support the findings of this study are available in the Supporting Information.
