# Supplementary material for: Structural Design and DLP 3D Printing Preparation of High Strain Stable Flexible Pressure Sensors
Source: Adv Sci (Weinh). 2023 Nov 12;11(37):2304409. doi: 10.1002/advs.202304409 (PMC11462308; doi:10.1002/advs.202304409)
Supplement: Supplementary file 1 — Supporting information [file ADVS-11-2304409-s001.pdf]

ADVANCED SCIENCE
Open Access

Supporting Information

for *Adv. Sci.*, DOI 10.1002/advs.202304409

Structural Design and DLP 3D Printing Preparation of High Strain Stable Flexible Pressure Sensors

Xiangling Xia, Ziyin Xiang, Zhiyi Gao, Siqi Hu, Wuxu Zhang, Ren Long, Yi Du, Yiwei Liu, Yuanzhao Wu, Wenxian Li, Jie Shang and Run-Wei Li**

Supporting Information

Structural Design and DLP 3D Printing Preparation of High Strain Stable Flexible Pressure Sensors

Xiangling Xia#, Ziyin Xiang#, Zhiyi Gao, Siqi Hu, Wuxu Zhang, Ren Long, Yi Du, Yiwei Liu, Yuanzhao Wu, Wenxian Li, Jie Shang, Run-Wei Li**

1. Strain Sensitivity Equation based on Linear Elasticity Theory

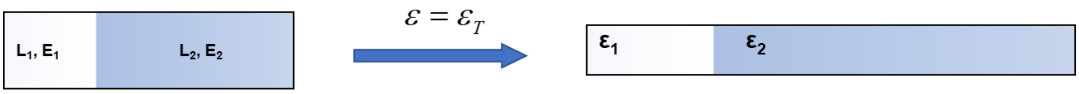

Figure S1 Two-dimensional simplified diagram of the force-sensitive structure

The traditional cylindrical force-sensitive structure of a sensor can be simplified as a two-dimensional geometric structure, as shown in **Figure S1**. In this structure, $\mathbf{L_1}$ and $\mathbf{L_2}$ represent the original lengths of the high modulus force-sensitive structure and the low modulus substrate, respectively. $\mathbf{E_1}$ and $\mathbf{E_2}$ represent the elastic modulus of the high modulus force-sensitive structure and the low modulus substrate, respectively. $\boldsymbol{\varepsilon_1}$ and $\boldsymbol{\varepsilon_2}$ represent the tensile strains of the high modulus force-sensitive structure and the low modulus substrate, respectively, while ε represents the overall tensile strain of the material.

Assuming that the material undergoes tensile strain and experiences uniform deformation within Materials 1 and 2, while neglecting any deformation mismatch at the interface of Materials 1 and 2, the following equations can be derived based on the linear elasticity theory and resistance calculation formula:

$$\varepsilon_1 E_1 \cdot h(1-\varepsilon_1\mu_1)\cdot r = \varepsilon_2 E_2 \cdot h(1-\varepsilon_2\mu_2)\cdot r \tag{1}$$

$$(\varepsilon_1+1)L_1+(\varepsilon_2+1)L_2=(\varepsilon_T+1)(L_1+L_2) \tag{2}$$

$$R=\rho\frac{l}{S}=\rho\frac{(1-\varepsilon_1\mu_1)h}{L_1(\varepsilon_1+1)\cdot(1-\varepsilon_1\mu_1)r}=\frac{\rho h}{(\varepsilon_1+1)L_1 r} \tag{3}$$

Here, **r** represents the depth of the two-dimensional geometric structure, **h** represents the initial height of the two-dimensional structure, **R** represents the resistance value of the structure, represents the resistivity of the material, **l** represents the length of the resistance region, and **S** represents the cross-sectional area of the resistance region.

When assuming that the material used is incompressible, its Poisson's ratio can be approximated as $\mu_1=\mu_2=0.49$. Under the small tensile strain condition $\varepsilon_1,\varepsilon_2<0.2$, $1-\varepsilon_{1,2}\mu_{1,2}\approx 1$, Equation (1) combined with Equations (2) and (3) yields:

$$R=\frac{\rho h}{L_1 r}\cdot\frac{L_1+M_r L_2}{\varepsilon_T(L_1+L_2)+L_1+M_r L_2}, M_r=\frac{E_1}{E_2} \tag{4}$$

From Equation (4), the tensile strain sensitivity of the structure, S_ε, can be expressed as:

$$\frac{\Delta R}{R_0}/\varepsilon = -\frac{L_1 + L_2}{(1+\varepsilon_T)L_1 + (\varepsilon_T + M_r)L_2} \tag{5}$$

$$S_\varepsilon = \left|\frac{\Delta R}{R_0}/\varepsilon\right| = \frac{L_1 + L_2}{(1+\varepsilon_T)L_1 + (\varepsilon_T + M_r)L_2} \tag{6}$$

From Equation (6), it can be observed that the tensile strain sensitivity, $\mathbf{S_\varepsilon}$, is negatively correlated with $\mathbf{M_r}$, indicating that a higher modulus of the force-sensitive structure leads to a lower response of the sensor to tensile strain. Additionally, when **L** (**L** = $\mathbf{L_1}$ + $\mathbf{L_2}$) is a constant, differentiating the sensitivity, $\mathbf{S_\varepsilon}$, with respect to $\mathbf{L_1}$ yields:

$$\frac{\partial S_\varepsilon}{\partial L_1} = \frac{(M_r - 1)L}{((\varepsilon_T + M_r)L + (1 - M_r)L_1)^2} \tag{7}$$

Indeed, when $M_r > 1$, it is evident that $\frac{\partial S_\varepsilon}{\partial L_1} > 0$. This implies that the tensile strain sensitivity, $\mathbf{S_\varepsilon}$, is positively correlated with $\mathbf{L_1}$, indicating that a smaller proportion of $\mathbf{L_1}$ results in a lower response of the sensor to tensile strain.

2. Finite Element Simulation and Optimization of Force-Sensitive Structures

Table S1 $L_{18}(3^7)$ orthogonal table

No.	n	rb	tb	Side	Mod	Dis	Cur
1	2	3	1	2	1	2	1
2	1	3	2	2	3	1	2
3	3	1	1	2	3	3	2
4	3	2	2	2	2	2	1
5	2	1	3	2	2	1	3
6	1	1	2	3	2	3	1
7	1	2	1	3	3	2	3
8	1	3	3	1	2	2	2
9	1	1	1	1	1	1	1
10	2	2	2	3	1	1	2
11	2	3	3	3	3	3	1
12	2	1	2	1	3	2	3
13	3	2	3	1	3	1	1
14	3	3	1	3	2	1	3
15	3	3	2	1	1	3	3
16	2	2	1	1	2	3	2
17	3	1	3	3	1	2	2
18	1	2	3	2	1	3	3

Table S2 Parameter values corresponding to each level

Level			1	2	3
Factor	**n**		1	2	3
	**rb**	**mm**	0.5	0.75	1
	**tb**		0.5	0.75	1
	**Side**		3	5	Inf
	**Mod (Yeoh)**	**C_1 (Pa)**	97674	514180	3.1588
		**C_2 (Pa)**	2618.3	1510400	-117.7
		**C_3 (Pa)**	371.16	-256.8	29272000
	**Dis (Array)**	**mm**	6 (3×3)	4 (4×4)	3 (5×5)
	**Cur**	**m^{-1}**	0	$1/3C_{max}$*	$2/3C_{max}$*

$$*C_{\max} = \frac{2H/n}{(1-tb)^2 rb^2 + (H/n)^2}$$

Table S2 displays the specific values corresponding to the levels of each factor, for example, **rb** Level 1 corresponds to 0.5mm. Each parameter is set to three levels: **MIN**, **MID**, and **MAX**, to ensure coverage of the parameter's variability. Taking into account the actual precision of 3D printing, some parameters have the following levels: stacking number **n** (1, 2, 3), bottom edge length **rb** (0.5mm, 0.75mm, 1mm), top-bottom ratio **tb** (0.5, 0.75, 1), and array spacing **Dis** (6mm, 4mm, 3mm). **Mod** parameter represents three types of resin with different amounts of hardener added, and three sets of Yeoh hyperelastic model parameters calculated from stress-strain curves after curing. The number of sides **Side** takes theoretical minimum value 3, mid-value 5, and maximum value Inf (meaning a polygon with infinite sides, i.e., a circle). The side curvature **Cur** can be calculated to obtain its maximum value C_{max}. However, when the curvature is at its maximum value, there is a tangential contact area between the force-sensitive structure and the elastic substrate that is challenging to achieve in the printing process. Therefore, it takes theoretical minimum value 0 (i.e., a straight line), $1/3C_{max}$, and $2/3C_{max}$.

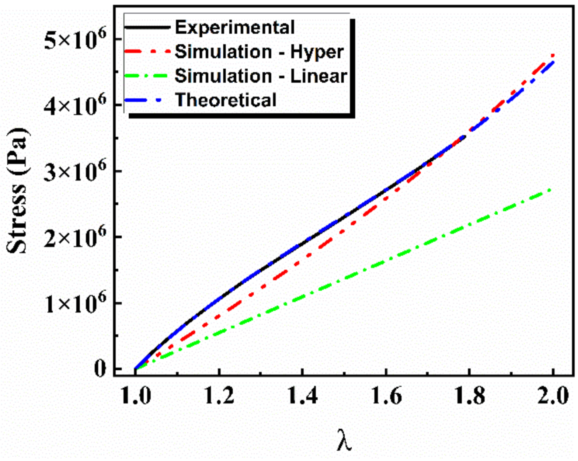

Figure S2 Verification of the COMSOL Finite Element Simulation Model.

To validate the finite element simulation model, we used COMSOL to create an ISO standard tensile specimen and stretched it to 200% of its original length (i.e., ε =100%). We compared the calculated stress values (including the hyperelastic and linear elastic models) with experimental and theoretical values, as shown in the graph below. From the graph, it is evident that the stress-strain theoretical values based on the Yeoh hyperelastic model (Theoretical) closely match the experimental values (Experimental), indicating that the mechanical parameters (c1, c2, c3) of the model were accurately calculated.

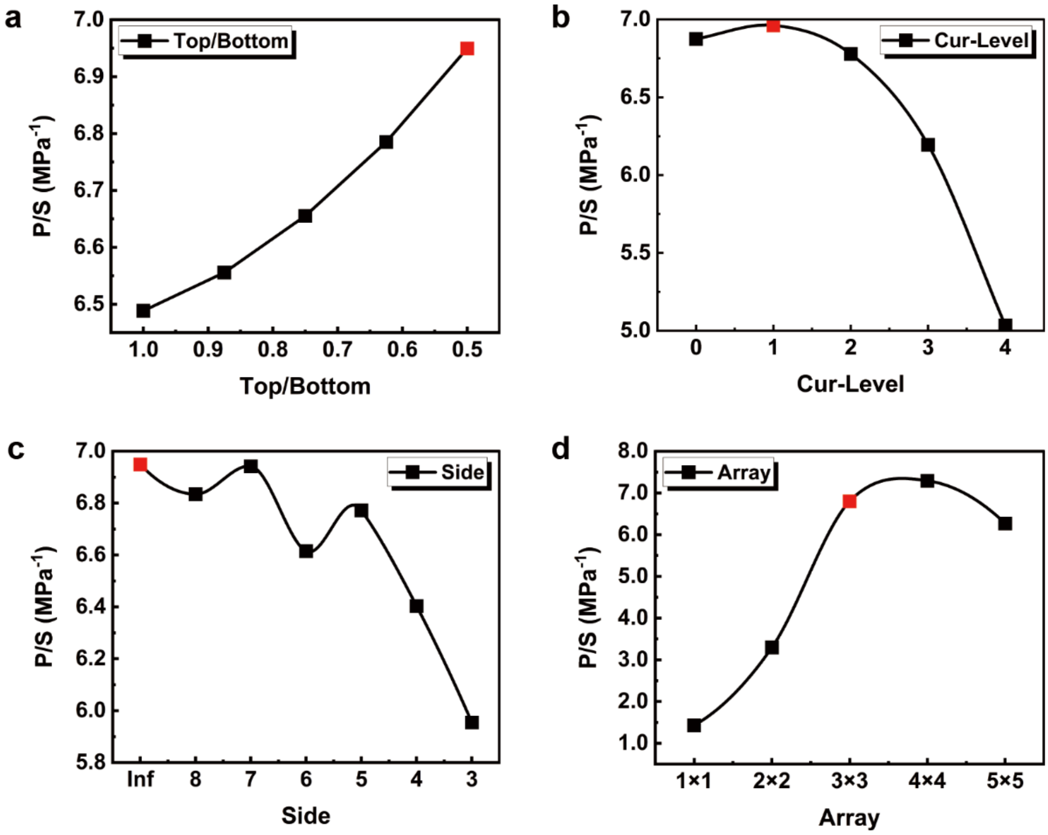

Figure S3 The relationship between the ratio of pressure sensitivity to tensile strain sensitivity (P/S) and structural parameters is shown in the following figures. The P/S ratio as a function of **(a)** aspect ratio, **(b)** curvature level, **(c)** number of edges, and **(d)** array density.

Figure S3 is a comprehensive consideration of pressure sensitivity and tensile strain sensitivity, the purpose is to select the optimal variable. By calculating the ratio of pressure sensitivity to tensile strain sensitivity, denoted as P/S, we can effectively evaluate the sensor's performance. A higher P/S value indicates a more desirable outcome, as it signifies superior pressure sensitivity relative to its sensitivity to tensile strain. It is worth noting here that, in Figure S2(d), although a 4x4 array offers the potential to maximize the P/S value, it was not selected in this study due to practical considerations and limitations imposed by the accuracy of the 3D printer employed. The high density of a 4x4 array posed challenges in terms of fabrication and limited the achievable accuracy. Consequently, a 3x3 array was chosen as a compromise, taking into account experimental constraints while still providing valuable insights into the sensor's performance.

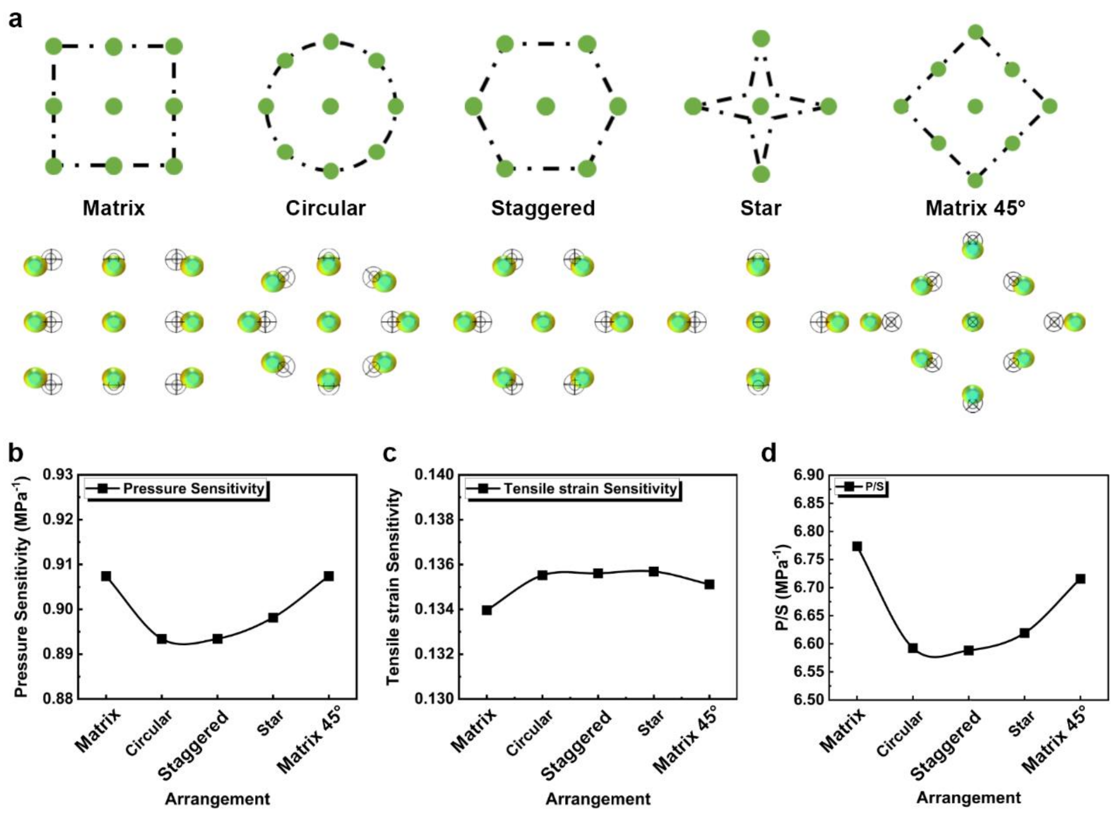

Figure S4 The impact of different distribution of the force sensitive structures on the strain sensitivity and pressure sensitivity of the sensor. (a) Distribution patterns (top) and displacement of the force-sensitive structure at 20% strain (black box represents the initial position). **(b)** Pressure sensitivity of the sensor corresponding to different distribution patterns. **(c)** Strain sensitivity of the sensor corresponding to different distribution patterns. **(d)** Ratio of pressure sensitivity to strain sensitivity for different distribution patterns.

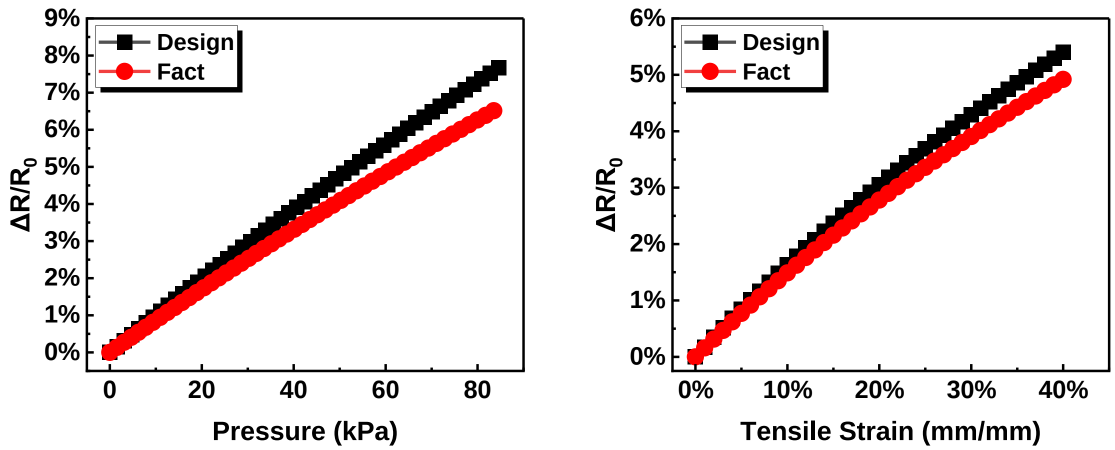

Figure S5 Finite element simulations of the designed structure and the fabricated structure. (a) Pressure-resistance change relationship. **(b)** Tensile strain-resistance change relationship.

3. Selection of Photosensitive Resin and Preparation of Conductive Resin

Table S3 Nominal parameters of flexible photopolymer resin

Resin	Viscosity mPa·s	Curing Hardness
Agilus 30	100	40 A
Are3D	2000	50 A
F39-T	980	60-75A
X23	980	75 D
X29	980	75 D

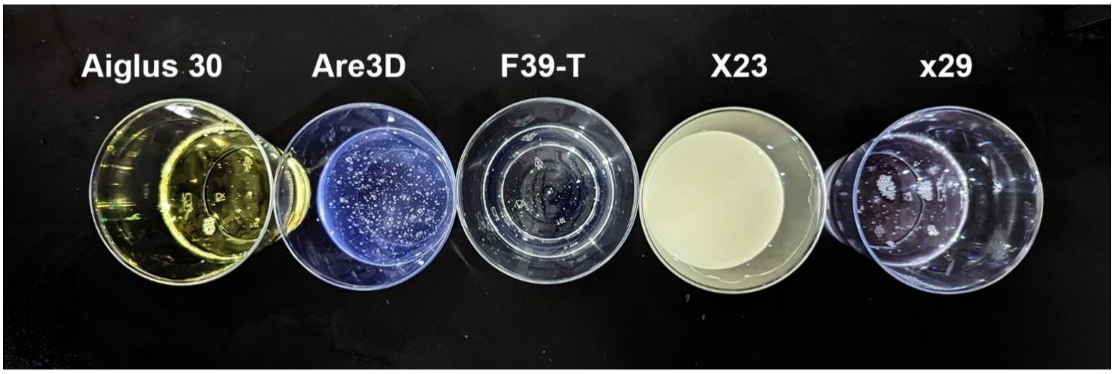

Figure S6 Pictures of the five resins: Agilus 30, Are3D, F39-T, X23 and X29

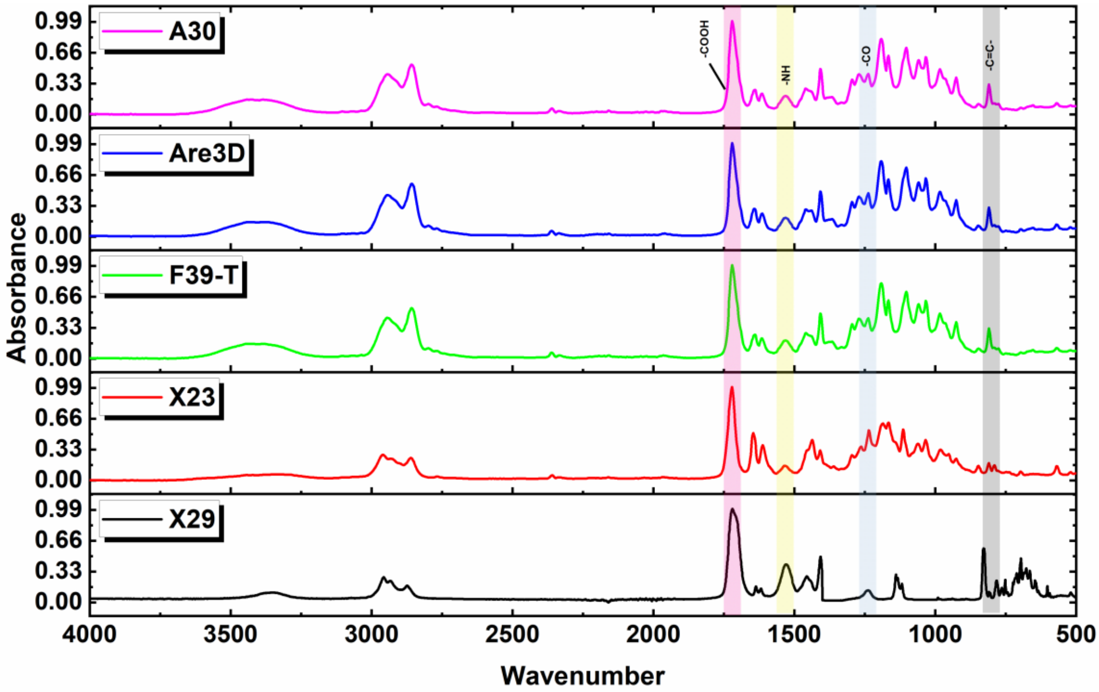

Figure S7 Infrared spectra of Agilus 30, Are3D, F39-T, X23, and X29 resins

We conducted a comparative study on the infrared spectra and mechanical properties of different types of flexible photosensitive resins. **Figure S7** shows the infrared spectra and cured infrared spectra of the five

resins. From the analysis of the infrared spectra, it can be observed that all the flexible UV-curable resins used in the experiment exhibit characteristic peaks of carbonyl C=O (1722-1726 cm^{-1}), C-O asymmetric and symmetric stretching vibrations (1275-1280 cm^{-1}, 1180-1197 cm^{-1}), indicating that they are polyacrylate-based resin materials.[1] Under the action of ultraviolet light, the photoinitiator triggers a chain polymerization reaction of the alkene bonds.

The process of photoinitiated polymerization is as follows[2]:

$$PI + hv \rightarrow PI*$$
$$PI* \rightarrow X\bullet + Other$$
$$X\bullet + CH_2 = CHCOOR \rightarrow X - CH_2 - \dot{C}HCOOR$$
$$X - CH_2 - \dot{C}HCOOR + nCH_2 = CHCOOR$$
$$\rightarrow XCH_2 - CHCOOR_n CH_2 - \dot{C}HCOOR$$

Among them, **PI** represents the photoinitiator, **hv** represents photons, **PI*** represents the excited state of the photoinitiator molecule, **X·** represents a free radical, represents an acrylate monomer, and **R** represents an arbitrary hydrocarbon group.

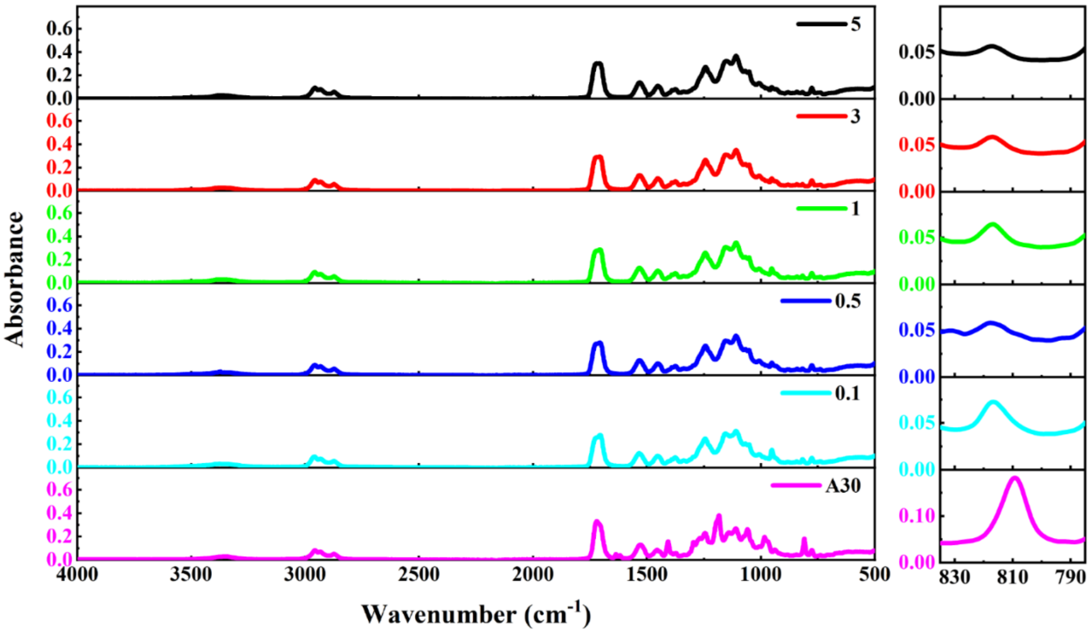

Figure S8 Infrared absorption spectra of cured products correspond ding to different AIBME contents (glass side), with an enlarged view of the 810cm^{-1} region

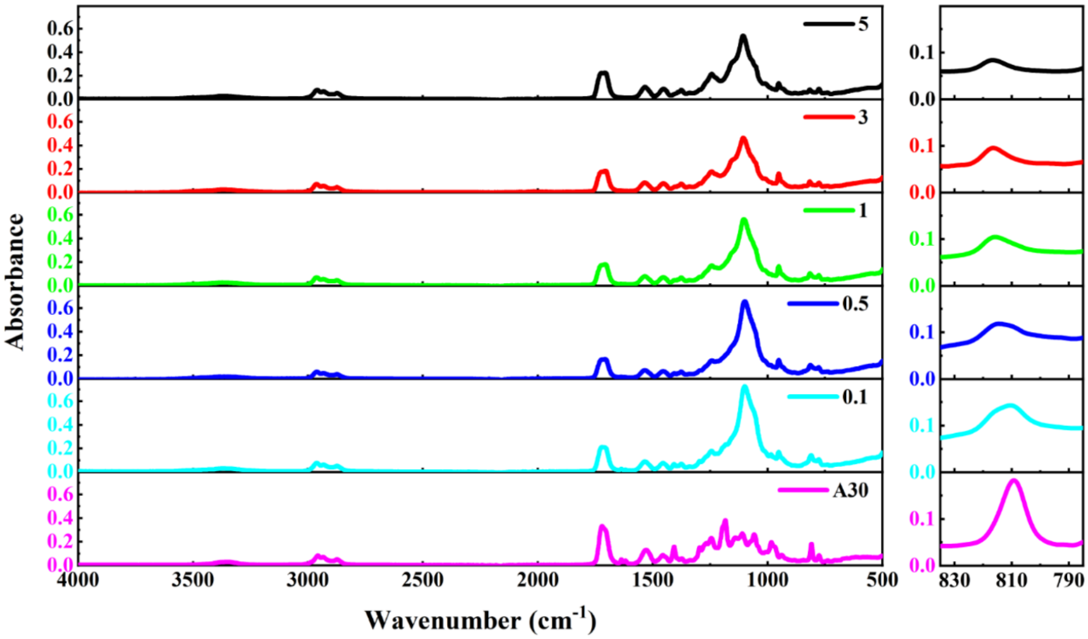

Figure S9 Infrared absorption spectra of cured products corresponding to different AIBME contents (PTFE side), with an enlarged view of the 810cm^{-1} region

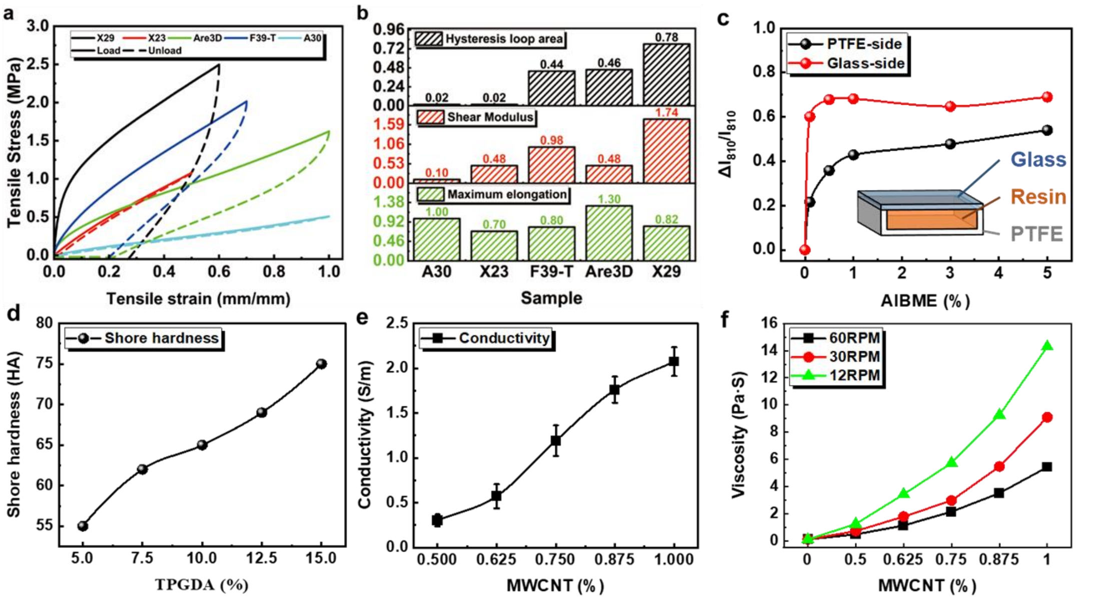

Figure S10 The performance of resin materials and the influence of additives on the mechanical and electrical properties of the resin. (a) Stress-strain curves of the tension specimens prepared from the five resin materials within their fracture elongation range. **(b)** Hysteresis loop area, shear modulus, and maximum elongation of the five resin materials. **(c)** Influence of AIBME content on the conversion rate of carbonyl groups. **(d)** Influence of TPGDA content on the hardness of the cured resin. **(e)** Influence of MWCNT content on the electrical conductivity of the cured resin. **(f)** Influence of MWCNT content on the viscosity of the resin slurry.

The stress-strain curves of the cured samples of the photosensitive resin were obtained through tensile testing (**Figure S10a, b**), and the material parameters were fitted using the Yeoh hyperelastic model.[3] The model equation is as follows:

$$\bar{I}_{1_{uni}} = \lambda^2 + \frac{2}{\lambda} \tag{8}$$

$$P_{1_{uniaxial}} = 2(\lambda - \frac{1}{\lambda^2})\sum_{p=1}^{3} pc_p(\bar{I}_{1_{uni}} - 3)^{p-1} \tag{9}$$

Where $\lambda = L / L_0$ is the stretch ratio, $c_p(p = 1, 2, 3)$ is the material parameter to be fitted, and $2c_1$ can be interpreted as the shear modulus.

The carbonyl conversion rates calculated from the infrared spectra are shown in **Figure S10c**. It can be observed from the graph that with an increase in AIBME content, the carbonyl conversion rates on both sides of the cured sample gradually increase. When the mass fraction of AIBME exceeds 1%, the carbonyl conversion rates on the glass side and PTFE side reach saturation. This indicates that a mass fraction of 1% AIBME is sufficient for complete curing of the A30 resin at 80°C for 1 hour.

The results of studying the effect of TPGDA content on material hardness are shown in **Figure S10d**. Clearly, as the TPGDA content increases, the hardness of the cured product also increases rapidly, indicating the need to increase the TPGDA content. However, on one hand, a high amount of TPGDA reduces the MWCNT content, leading to a decrease in the conductivity of the material. On the other hand, excessively high hardness results in a rapid decrease in the pressure sensitivity of the sensor, which contradicts the application goal of pressure sensors. Considering the trade-off between conductivity, pressure sensitivity, and hardness, a TPGDA content of 10 wt% was chosen, resulting in a hardness of 65A for the cured resin.

In the study, different mass fractions of MWCNT were mixed with A30A slurry to obtain conductive filling slurry (A30-A-MC). Due to the low density of MWCNT, increasing its mass fraction to achieve electrical conductivity often leads to a rapid decrease in the flowability of the slurry and an increase in its viscosity. **Figure S10e** shows the relationship between the electrical conductivity of the cured material and MWCNT content in the slurry, and **Figure S10f** shows the variation of viscosity of A30-A-MC slurry with increasing MWCNT content. It is evident that increasing the MWCNT content enhances the conductivity of the material but also significantly increases the viscosity of the slurry, showing shear-thinning behavior. Therefore, an appropriate value of MWCNT content should be chosen to facilitate the free flow and filling of the conductive slurry. In the experiments, it was found that a MWCNT content of 0.75% provides good electrical conductivity (1.2 S/m) and satisfactory flowability (2.14 Pa·s), making it suitable for filling the substrate.

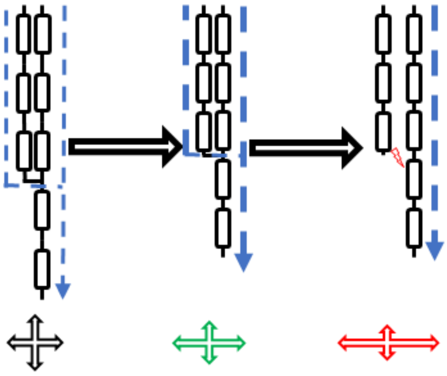

Figure S11 The branch resistance network model.

This equivalent circuit consists of a main path and a branch path. In the initial stages of stretching, both the main path and the branch path remain connected. Due to the Poisson effect, the distance in the vertical direction of the conductive network shortens, resulting in a decrease in resistance. However, when the stretching exceeds a certain degree, the branch path disconnects from the main path, causing an overall increase in resistance in the circuit. Therefore, during the actual stretching process, the phenomenon of resistance decreasing and then increasing occurs. This effect is challenging to model through finite element simulations, so the finite element simulation in this study did not introduce this effect and only considered the influence of strain on resistance.

References

[1] a) A. Dong, T. Wan, S. Feng, D. Sun, *Journal of Polymer Science Part B: Polymer Physics* **1999**, *37* (18), 2642, https://doi.org/https://doi.org/10.1002/(SICI)1099-0488(19990915)37:18<2642::AID-POLB8>3.0.CO;2-D; b) L. Balan, R. Schneider, D. J. Lougnot, *Progress in Organic Coatings* **2008**, *62* (3), 351, https://doi.org/https://doi.org/10.1016/j.porgcoat.2008.01.017; c) C. Decker, K. Zahouily, *Polymer Degradation and Stability* **1999**, *64* (2), 293, https://doi.org/https://doi.org/10.1016/S0141-3910(98)00205-5.

[2] a) J. Z. Manapat, Q. Chen, P. Ye, R. C. Advincula, *Macromol. Mater. Eng.* **2017**, *302* (9), 1600553, https://doi.org/10.1002/mame.201600553; b) C. Peinado, N. S. Allen, E. F. Salvador, T. Corrales, F. Catalina, *Polymer Degradation and Stability* **2002**, *77* (3), 523, https://doi.org/https://doi.org/10.1016/S0141-3910(02)00111-8.

[3] O. H. Yeoh, *Rubber Chem. Technol.* **1993**, *66* (5), 754, https://doi.org/10.5254/1.3538343.
